# Development and Characterization of an Ethyl Methane Sulfonate (EMS) Induced Mutant Population in *Capsicum annuum* L.

**DOI:** 10.3390/plants9030396

**Published:** 2020-03-23

**Authors:** Muhammad Irfan Siddique, Seungki Back, Joung-Ho Lee, Jinkwan Jo, Siyoung Jang, Koeun Han, Jelli Venkatesh, Jin-Kyung Kwon, Yeong Deuk Jo, Byoung-Cheorl Kang

**Affiliations:** 1 Department of Plant Science and Plant Genomics and Breeding Institute, Seoul National University, Seoul 08826, Korea; arafay68@yahoo.com (M.I.S.); sseung1201@snu.ac.kr (S.B.); shm97257@gmail.com (J.-H.L.); jingonak@gmail.com (J.J.); hoifirstlove@snu.ac.kr (S.J.); hke1221@snu.ac.kr (K.H.); venkatesh_jelli@yahoo.com (J.V.); kwonjk90@hanmail.net (J.-K.K.); 2 Radiation Breeding Team, Advanced Radiation Technology Institute, Korea Atomic Energy Research Institute, Jeongeup 56212, Korea; jyd@kaeri.re.kr

**Keywords:** EMS, pepper, mutation breeding, TILLING, phenotyping

## Abstract

Plant breeding explores genetic diversity in useful traits to develop new, high-yielding, and improved cultivars. Ethyl methane sulfonate (EMS) is a chemical widely used to induce mutations at loci that regulate economically essential traits. Additionally, it can knock out genes, facilitating efforts to elucidate gene functions through the analysis of mutant phenotypes. Here, we developed a mutant population using the small and pungent ornamental *Capsicum annuum* pepper “Micro-Pep”. This accession is particularly suitable for mutation studies and molecular research due to its compact growth habit and small size. We treated 9500 seeds with 1.3% EMS and harvested 3996 M_2_ lines. We then selected 1300 (32.5%) independent M_2_ families and evaluated their phenotypes over four years. The mutants displayed phenotypic variations in plant growth, habit, leaf color and shape, and flower and fruit morphology. An experiment to optimize Targeting Induced Local Lesions IN Genomes (TILLING) in pepper detected nine EMS-induced mutations in the *eIF4E* gene. The M_2_ families developed here exhibited broad phenotypic variation and should be valuable genetic resources for functional gene analysis in pepper molecular breeding programs using reverse genetics tools, including TILLING.

## 1. Introduction

Pepper (*Capsicum annuum* L.) is an economically important crop worldwide. Peppers are consumed as food for their richness in nutritional and medicinal substances, including capsaicinoids, carotenoids, and vitamins A and C. They are also used in the cosmetic and pharmaceutical industries, and for ornamental purposes [1,2]. Breeding of modern pepper cultivars focuses on improving economically important traits, including yield and nutrient and secondary metabolite content [3,4,5,6]. These programs require vast genetic diversity, which can be promoted by mutagenesis to alter nucleotide sequences, potentially creating novel alleles useful for crop improvement [7,8]. There are four main categories of agents used to alter the genome: physical mutagens (e.g., radiation with X-rays, gamma-rays, fast neutrons, or UV); chemical agents (e.g., ethyl methane sulfonate (EMS), ethyl nitrosourea (ENU), 1,2:3,4-diepoxybutane (DEB), and N-nitroso-N-methylurea (NMU); biological agents (e.g., T-DNA and transposons); and genome editing (e.g., Transcription Activator-Like Effector Nucleases (TALENs) and Clustered, Regularly Interspaced, Short Palindromic Repeats CRISPR-Cas9) [8,9,10].

Physical mutagens have been used to produce mutant populations in pepper [4,11,12]. However, gamma radiation and fast neutrons can induce larger DNA inversions and deletions that hinder the identification of the genes underlying a mutant phenotype [8]. As an alternative, EMS, a chemical agent, has been commonly applied to induce mutations in seeds. EMS induces random point mutations at a high frequency, some of which can create novel stop codons in genes of interest [13,14]. EMS has been successfully used in the Solanaceae family to generate morphological diversity and drive the improvement of desirable traits, including yield, fruit quality, disease resistance, and male sterility. EMS-derived mutations in the tomato (*Solanum lycopersicum*) eukaryotic initiation factor 4E (*eIF4E*) confer resistance to potyviruses [15,16]. A screen in a mutant eggplant (*Solanum melongena*) population recovered various alleles with increased content of phenolic compounds (i.e., anthocyanin and chlorogenic acid (CGA) [17]. In pepper, multiple studies have used mutant populations obtained via EMS mutagenesis [7,18,19,20,21]. In particular, an EMS mutant population of the sweet pepper cultivar “Maor” was created to investigate the genes that regulate flower and plant architecture [12,22]. According to the official data published by the FAO/IAEA program (http://mvd.iaea.org), 16 pepper cultivars have been bred through mutagenesis technology so far.

Populations derived from EMS mutagenesis can be analyzed through forward or reverse genetics. Technologies like high-resolution melting (HRM) and Targeting Induced Local Lesion IN Genome (TILLING) can identify EMS-induced point mutations. TILLING is a high-throughput, non-transgenic, reverse genetics method used in mutagenized populations to discover novel allelic variations at known loci [23,24]. By leveraging these approaches, novel alleles of the target loci can be quickly identified and become accessible to ongoing breeding programs [25]. TILLING relies on the potential of the mutagen to induce abundant point mutations that generate novel SNPs throughout the genome. It was employed successfully to uncover mutations in genes for important agronomical traits in Arabidopsis, maize, rice, and tomato [26]. 

Whereas it is possible to detect dominant mutations in the M_1_ generation, most induced alleles are recessive and will cause observable phenotypes only in the M_2_. Nevertheless, the efficiency of the mutagenesis can be hinted in the M_1_ by the presence of phenotypes like chlorophyll defects (e.g., albino plants), pollen sterility, dwarfism, curled leaves, and early or late flowering [21]. As the mutations will usually segregate in the M_2_ population to produce homozygotes for dominant or recessive alleles [27], visual screening and characterization can be highly effective in detecting mutant phenotypes at that stage. Desirable characteristics, including changes in the growth period, plant height, fruit color, disease and pest resistance, or flowering time, can be used as markers to select candidate mutants [21,28]. 

Pepper breeding for improvement of various agronomic traits including disease and pest resistance is hindered due to the limited genetic diversity. Mutagenesis can expeditiously produce genetic variability in both qualitatively and quantitatively inherited traits in crops. In tomato, mutant libraries are available in various backgrounds [29,30,31,32], whereas mutant research in pepper is still relatively limited. Furthermore, TILLING also has not been optimized for this species. Therefore, in the present study we constructed a mutant population using a dwarf, compact, and easy-to-grow pepper accession, Micro-Pep, using EMS and characterized the phenotypes of the resulting population and optimized the TILLING protocol by identifying mutations in the *eIF4E* gene. The mutant population developed in this study will be useful to identify novel genes for pepper breeding and will serve as a platform for forward and reverse genetics studies. 

## 2. Material and Methods

### 2.1. Plant Material

The Micro-Pep pepper (*C. annuum* L.), which has a dwarf and compact growth habit, was used to create a mutant population by EMS. The seeds were surface disinfected with 10% trisodium phosphate for 20 min, followed by 50% Clorox for 10 min, and then washed under tap water for 1 h. 

### 2.2. Mutagenesis

The concentration of EMS to be used was optimized prior to the large-scale treatment: seeds (100 per treatment) were treated with 1.0, 1.3, 1.5, or 2.0% of EMS and their germination percentage quantified. For the large-scale experiment, 9500 seeds were mutagenized in four batches. Seeds were presoaked in distilled water in an orbital shaking incubator (100 rpm) for 18 h at 24 °C. Seeds were then immersed in a 1.3% EMS (Sigma-Aldrich, Saint Louis, MO, USA) solution in 0.1 M sodium phosphate buffer (pH 7.0) to prevent rapid hydrolysis [19] and incubated for 12 h at 20℃ in a shaking incubator (100 rpm) in a dark. Treated seeds were washed with 0.5% ethyl acetate (Sigma-Aldrich, Saint Louis, MO, USA) diluted in 0.1 M phosphate buffer (pH 7.0) for 50 min [7]. In a next step, seeds were washed with 0.5 mM sodium thiosulfate (pH 9.0) for 45 min. Finally, seeds were washed under running tap water for 3 h. Control seeds were treated with 0.1 M phosphate buffer (pH 7.0) and then processed like the EMS-treated seeds.

### 2.3. Construction of M_1_, M_2_, and M_3_ Generations

Micro-Pep M_1_ seeds were sown in 72-hole trays in soilless medium (Baroker-Seoul Bio, Korea). Seedlings were transplanted into small pots 30 days post-planting. M_1_ plants were observed, and their phenotypic data were recorded. After fruit ripening, the mature seeds were harvested, sorted, labeled, air dried, packed, and stored at the seed storage bank of the Seoul National University, Seoul, Korea in 2015. Ten plants each from 1300 M_2_ families were grown under greenhouse conditions. Standard agronomic practices were utilized, and the M_2_ generation was screened morphologically in the four-year interval from 2016 to 2019. The seeds were harvested in bulk for each M_2_ line and stored for the M_3_ generation. 

### 2.4. Phenotype Evaluation of M_2_ Plants

M_2_ plants were evaluated for plant growth, habit, leaf color and shape, and flower- and fruit color, morphology-related traits. Each M_2_ line was evaluated for growth abnormalities from seedling to fruit harvesting. The phenotypes were recorded according to four major classes and eleven subclasses [7]. Furthermore, the frequency and segregation ratio of mutant characters in each M_2_ line was calculated.

### 2.5. TILLING Experiment

DNA of each M_2_ plant was extracted from 3-week-old seedlings as described previously [3] and bulked (10×) to make one representative sample for each M_2_ family. Seven-hundred M_2_ samples of Micro-Pep and 1760 Yuwol-cho samples [3,7] comprising 2460 M_2_ mutant families were used to optimize the TILLING protocol for pepper. Wild-type Micro-Pep, Yuwol-cho, and two other *C. annuum* accessions (i.e., CM334 and Dempsey) were used as controls. The DNA of all the samples and control accessions was normalized at 20 ng/μL using sterile distilled water and pooled four-fold. Primers were designed for the *eIF4E* sequence (*C. annuum pvr1* locus (accession No. NC_029980.1)) with the Primer3 web tool (Table 1). PCR amplification was performed as previously described by [3]. TILLING was performed in four steps: (1) PCR amplification with gene-specific primers, (2) heteroduplex DNA construction, (3) cleavage by endonucleases using the mutation detection Kit-S1000 (Integrated DNA Technologies, Inc. USA), and (4) mutation detection by capillary gel electrophoresis analysis (Advanced Analytical Technologies, Inc., Ankeny, IA, USA). The Sanger sequencing method was used to evaluate the results obtained in the enzymatic mismatch cleavage inspection.

## 3. Results

### 3.1. Effect of EMS dose on Seed Germination

**Figure 1:**
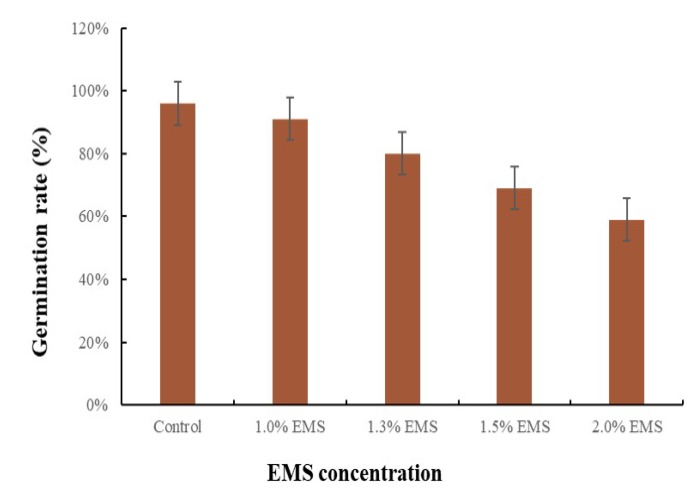
Germination rate in response to different ethyl methane sulfonate (EMS) concentrations. Frequency distribution of the effect of the concentration of EMS (%) on the germination rate at four different dosages (1, 1.2, 1.5, and 2%). Error bars represent the standard deviation of three replicates.

Micro-Pep is a decorative pepper plant with short internodes, whorled phyllotaxy, lanceolate leaves, and attenuate rounded fruits. Its compact growth and small size make the accession suitable for mutation studies. The EMS concentration used in the treatment of seeds is crucial for the successful development of a mutant population. Therefore, we tested the effect of different EMS concentrations (1, 1.3, 1.5, and 2% EMS) on germination two weeks after placing the seeds in moist petri dishes. As expected, the percentage of germinated seeds decreased with the increase in EMS concentration (Figure 1). The lowest germination (59%, compared with 96% in the control) was obtained with a treatment with 2% EMS, and the highest (91%) with 1% EMS (Figure 1). The germination markedly decreased from 80 to 69% with 1.3 and 1.5% of EMS, respectively (Figure 1). Based on these results, to ensure that we obtain a sufficient population after mutagenesis, we selected the 1.3% concentration to generate a mutant pepper population.

### 3.2. Phenotypes of M_1_ Mutant Plants

Of the 9500 seeds treated with EMS, 6620 (70%) germinated on medium (Table 2). We transplanted 4210 seedlings to pots with soil, scored their phenotypes, and harvested the seeds of the 3996 that reached maturity, for analysis in the M_2_ generation (Table 2). The M_1_ seedlings included mutants with aberrant phenotypes for plant height, foliage color, and inflorescence development (Table 3). In terms of plant height, 45 and 18 plants were scored as tall (>17 cm) and short (<10 cm) mutants, respectively (Table 3). The average height of normal Micro-Pep was 12.5 cm. We observed chimeras in the M_1_ population with albino or variegated foliage. Overall, 23 mutant individuals had foliage discoloration (Table 3). Flowerless mutants were the most abundant mutant category in the M_1_ generation, with 231 individuals displaying only vegetative growth (Table 3). Male sterility was also observed: 41 individuals had male-sterile flowers (Table 3). This abundance of mutant phenotypes confirmed that mutagenesis was successful and implied that a high number of mutants would be recovered in the subsequent M_2_ generation. 

### 3.3. Characterization of M_2_ Mutant Phenotypes

**Figure 2:**
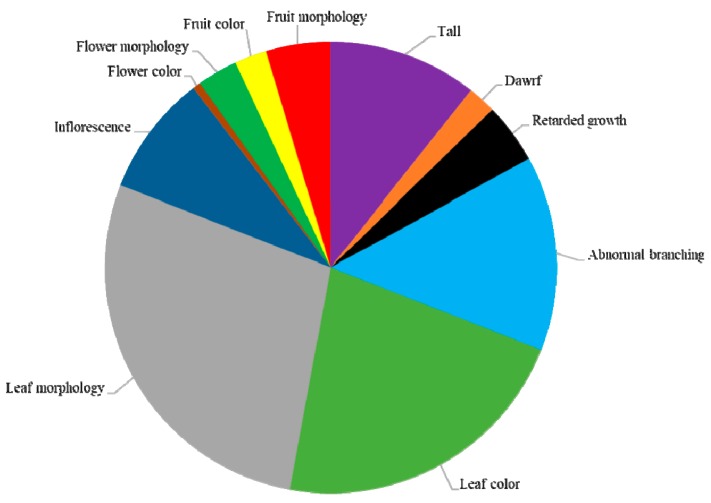
Classification of visible mutant phenotypes. Eleven subcategories and number of observed phenotypes in each category in the M_2_ generation.

**Figure 3:**
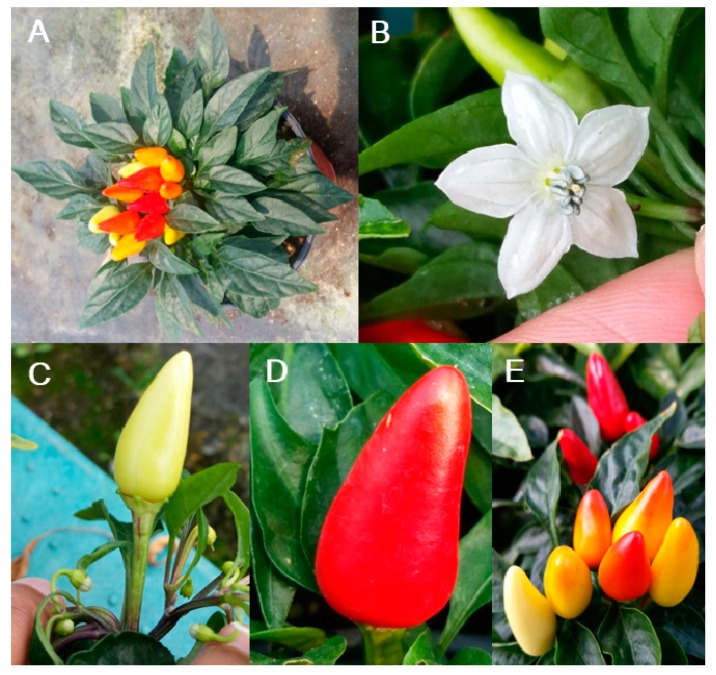
Phenotypes of non-treated EMS Micro-Pep plants. (**A**) Whole plant, (**B**) flower, (**C**) immature fruit, (**D**) mature fruit, and (**E**) fruit color at different stages.

Of the 1300 M_2_ lines selected for phenotypic analysis, 372 showed mutant phenotypes, which corresponded to a mutation frequency of 28.6% (Table 4). We divided the phenotypic alterations into four main classes: plant growth and habit, leaf color and morphology, flower characteristics, and fruit color and morphology (Figure 2 and Table 4). These main mutant categories included 11 subclasses (Figure 2). Mutants were named according to their phenotype category and compared with the wild-type Micro-Pep plants (Figure 3). Among the mutants, 30.5% had variations in plant growth and habit, 50.2% in leaf color and morphology, 12.3% in flower characteristics, and 7% in fruit color and morphology (Figure 2 and Table 4). Most of the mutations showed recessive inheritance, and many plants had pleiotropic phenotypes, in which individuals were altered in more than one trait (e.g., flowerless and dwarf individuals). 

### 3.4. Plant Growth and Habit Phenotypes

**Figure 4:**
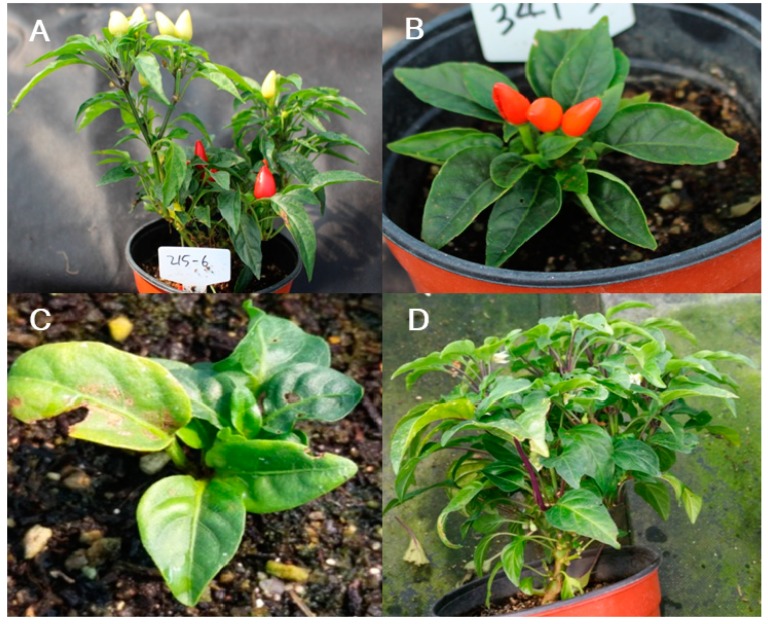
Plant height and growth mutant phenotypes. (**A**) Tall, (**B**) dwarf, (**C**) retarded growth, and (**D**) abnormal branching phenotypes.

The most conspicuous mutant class observed in the M_2_ generation was plant growth and habit (Figure 4). The mutant phenotypes included in this category were: aberrant plant height (tall or dwarf when compared with wild-type plants) (Figure 4A,B); retarded growth with no visible stem (Figure 4C); and abnormal branching with a multi-whorled canopy (Figure 4D). Of the mutant lines in the plant growth and habit, 37 were dwarves, 7 were tall, 15 had retarded growth, and 48 had abnormal branching (Table 4). These mutants can be used to explore the genetic mechanism underlying plant height in pepper breeding programs. 

### 3.5. Leaf Color and Morphology Phenotypes

**Figure 5:**
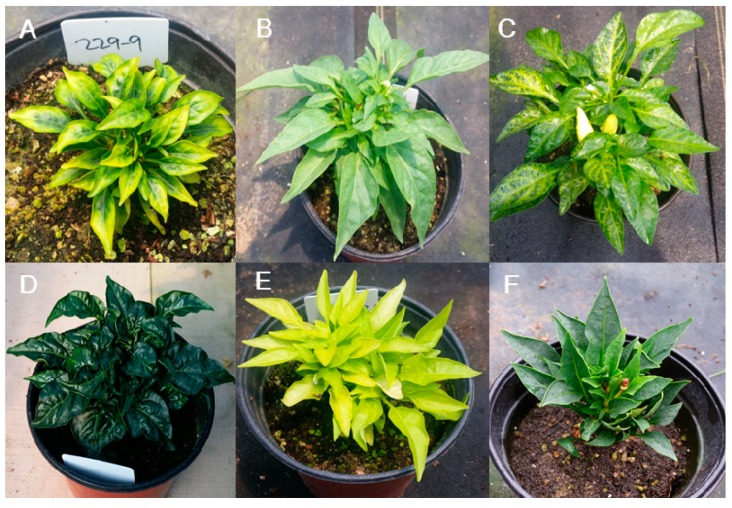
Leaf-color mutant phenotypes. (**A**) Mottled pale-green, (**B**) light-green, (**C**) mottled-yellow, (**D**) dark-green, (**E**) pale-green, and (**F**) silver-green leaves.

**Figure 6:**
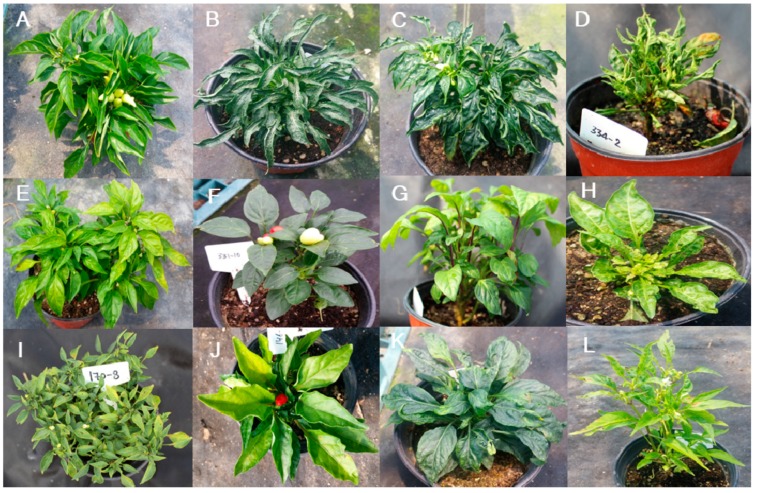
Leaf morphology mutant phenotypes. (**A**) Leathery texture, (**B**) undulation type, (**C**) upward curling, (**D**) downward curling, (**E**) bushy type, (**F**) obovate shape, (**G**) long petiole, (**H**) fused, (**I**) prolific, (**J**) large, (**K**) scabrous, and (**L**) narrow leaves.

The category with the highest number of mutants was leaf color and morphology (Figure 2; Table 4). We divided the leaf-color mutants into six types: mottled pale-green, light-green, mottled-yellow, dark-green, pale-green, and silver-green types. These types comprised 18, 6, 7, 13, 29, and 3 M_2_ mutant lines, respectively (Figure 5A–F; Table 4). For leaf morphology, we determined 12 subcategories with a variable number of mutant lines: leathery leaves (8); undulation type (5), upward curling (7), downward curling (4), bushy type (3), obovate shape (2), long petiole type (22), fused type (1), prolific type (11), large size (19), scabrous type (12), and narrow leaves type (9) (Figure 6A–L; Table 4). These mutants could be highly useful resources to study the genes involved in leaf color and morphology. 

### 3.6. Flower Color and Morphology Phenotypes

**Figure 7:**
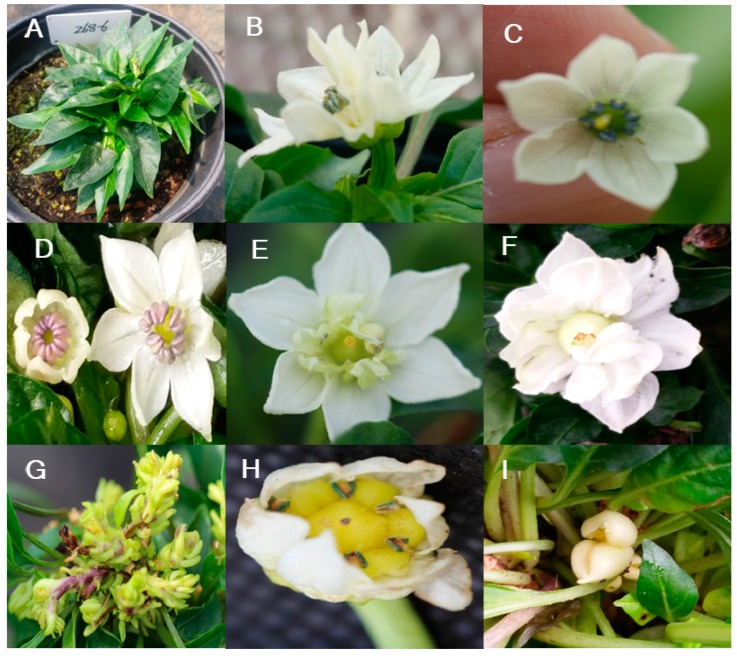
Flower color and morphology mutant phenotypes. (**A**) Flowerless, (**B**) twin flower, (**C**) sterile flower, (**D**) pink anthers, (**E**) filamentous stamen, (**F**) AGAMOUS type, (**G**) fasciculation type, (**H**) swelled ovary, with short stamen, and (**I**) shell type.

We observed three categories of mutants related to flower characteristics: inflorescence, organ color, and morphology (Table 4). Inflorescence mutants included 3 with twin flowers, 27 flowerless, and 21 sterile lines (Figure 7A–C; Table 4). A unique mutation related to flower organ color rendered the anthers pink (Figure 7D; Table 4). M_2_ lines with altered flower morphology included one filamentous-stamen, four AGAMOUS-type, one fasciculation-type, three with swelled ovary with short stamen, and one shell mutant (Figure 7E–I; Table 4). These flower mutants can be used to study the genes involved in pepper flower morphology and organ development. 

### 3.7. Fruit Color and Morphology

**Figure 8:**
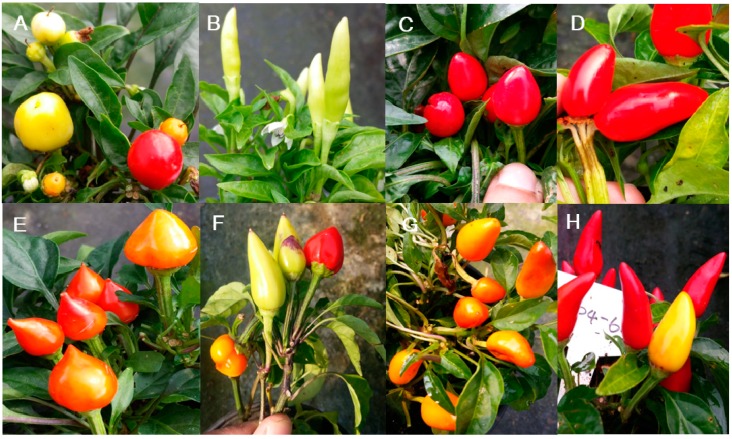
Fruit color and morphology mutant phenotypes. (**A**) Round shape, (**B**) cylindrical shape, (**C**) oval shape, (**D**) multiple fruit on a single calyx, (**E**) pyramid shape, (**F**) two shapes on one plant, (**G**) orange mature color, and (**H**) two different mature colors (red and yellow).

Several different types of mutations affected fruit morphology and color (Figure 8; Table 4). For morphology, we separated mutants according to alterations in fruit shape: round fruit, cylindrical shape type, oval shape, multi-fruit on single calyx, pyramid shape, and two fruit shapes on one plant. Those were present in 5, 2, 3, 1, 4, and 1 mutant lines, respectively (Figure 8A–F; Table 4). In contrast to the wild-type Micro-Pep fruits, which are red at maturity, seven mutant lines carried orange fruits, and one line had yellow and red fruits on the same plant (Figure 8G–H; Table 4). These mutants related to fruit shape represent potential breeding material to alter pepper fruit shape, as well as being resources to help elucidate the underlying genetic mechanisms. 

### 3.8. Mutation Screening and Detection by TILLING

**Figure 9:**
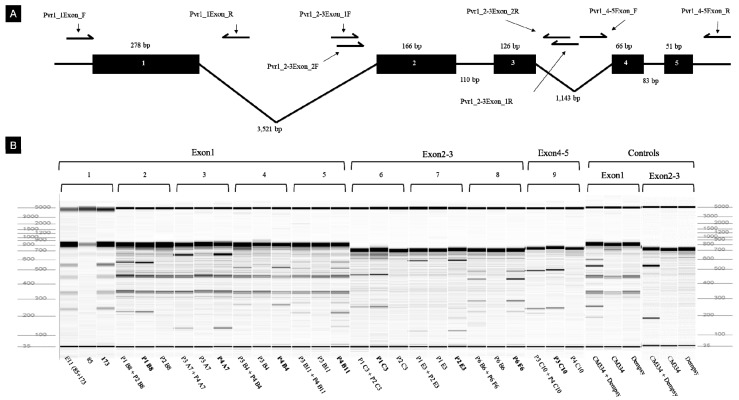
Mutations detected by Targeting Induced Local Lesions IN Genomes (TILLING). (**A**) Genomic structure of the *eIF4E* gene and primer positions. Black boxes show exons, and black lines depict introns. Arrows indicate the positions of each primer. (**B**) M_2_ lines of Micro-Pep, Yuwol-cho, and controls. The 1 and 8 lanes show tentative mutations detected in Micro-Pep M_2_ lines in exon 1 and exon 2-3, respectively. Lanes 2, 3, 4, and 5 represent mutations detected in exon 1; lanes 6 and 7 show mutations in exon 2-3; and lane 9 shows mutations in exon 4-5 of Yuwol-cho M_2_ lines. The mutated samples are highlighted with bold letters.

We next examined the suitability of TILLING analysis for mutant pepper populations, and tested this method to the *eIF4E* gene of Micro-Pep and Yuwol-cho M_2_ lines. This analysis revealed nine putative mutations (Figure 9; Table 5), which were confirmed by Sanger amplicon sequencing (Table 5). The *eIF4E* gene structure in pepper consists of five exons and four introns (Figure 9A). Two of the mutations were in Micro-Pep pooled samples, whereas seven were in Yuwol-cho (Table 5; Figure 9B). Five of the mutations localized to ‘Exon1’, three were detected in ‘Exon2-3’, and one was in ‘Exon 4-5’ (Figure 9B; Table 5). We confirmed the presence of single nucleotide mutations and their positions at the base-pair level (Table 5). The confirmed mutations all represented G:C to A:T base-pair transitions. These results confirmed that TILLING is a suitable method to find mutations in important pepper genes. Hence, the population developed in this study can serve in the future as an inclusive platform for reverse genetics studies in pepper.

## 4. Discussion

Mutagenesis by EMS is an effective approach to create genetic diversity in plant populations. In this study, we treated the dwarf *Capsicum annuum* L. accession Micro-Pep with 1.3% EMS and characterized the phenotypes of the M_1_, M_2_, and M_3_ generations. We analyzed 1300 M_2_ families and observed copious mutant phenotypes. A total of 372 families (28.6%) had clear mutations that affected the important traits of plant height, plant habit, leaf color, leaf morphology, and flower and fruit-related features.

Genetic diversity is fundamental to the success of breeding programs. Hence, it is imperative to maximize the effectiveness and efficiency of the mutagens used to generate that diversity [8,21]. The effect of a mutagen depends both on the concentration applied and on the germplasm treated. Therefore, it is necessary to optimize the procedure to assure a high mutation frequency without compromising seed viability [8,33]. This optimization is essential for EMS because a high concentration drastically reduces seed germination in multiple species [13]. The optimum dosage of EMS for rice, soybean, and tomato is below 1% [13,21,34] but it is higher (1.5%) for some pepper cultivars [7,35]. Here, we started by testing the effect of different EMS concentrations on Micro-Pep germination. Then, we treated bulk seeds with 1.3% EMS, which allowed 80% of germination and produced a mutation frequency high enough to create multiple mutants with observable phenotypes. 

Mutant phenotypes are less likely to emerge in the M_1_ generation, when only dominant mutations can be identified [36]. We identified several mutant phenotypes in M_1_ plants, including seedling growth defects, changes in plant height, alteration in foliage color, absence of inflorescences, and sterility. Previous mutation studies in pepper and eggplant also reported mutant phenotypes in M_1_ plants [17,18], though some of those were not present in subsequent generations. [35] and [21] similarly reported that many of the mutants observed in M_1_ pepper seeds mutagenized with EMS were not identified in the next generations. The mutation frequencies observed in the M_1_ generation may fluctuate substantially in M_2_ or M_3_ due to the non-heritability of large deletions that occur during mutagenesis [12].

M_2_ families of mutagenized populations reveal the recessive mutations and can show marked trait variation [17,21]. In accordance, the M_2_ generation of our study displayed striking mutant phenotypes with variable mutation frequencies. As seen in previous studies [17,37], plant height-related mutations were the most frequent. Dwarf mutants are fundamental for the elucidation of the regulatory mechanisms behind plant growth and development. They are also crucial for breeding of lodging-resistant cultivars [8]. We identified several dwarf mutants with abnormal branching, shorter internodes, or retarded growth with no obvious stems. Other dwarf mutants in pepper [4,7,21,38] are likely caused by the suppression of epidermal cell expansion or defects in gibberellin (GA) biosynthesis [39]. Identification of new recessive, monogenic mutations causing dwarfism could lead to the discovery of novel genes responsible for pepper growth and inform modern breeding programs [40].

We also identified chlorophyll mutants among the M_2_ lines, with a range of albino, yellow, pale-green, and dark-green phenotypes. The presence of chlorophyll mutants is a good indicator of the effectiveness of a mutagen in pepper [14], and EMS-treated pepper populations often display chlorotic and whitened leaves [7,21]. Here, the chlorotic mutants showed a range of mottled yellow and pigmented patterns. Mutants with leaf-color alterations are valuable for discovering genes responsible for chlorophyll metabolism [41]. Additionally, can be used as indicators of seed purity in the breeding of new cultivars in ornamental plants with better photosynthetic efficiency [42]. The leaf-color mutants identified here can provide alternative foliage color choices for breeders of ornamental peppers. 

We also observed mutants altered in leaf morphology, causing long petioles, and scabrous or proliferate leaves. In addition, we isolated several mutants with upward or downward curled leaves. Leaf curling can improve light energy absorption and photosynthetic rate. In addition, it can modulate leaf transpiration and enhance drought tolerance. Therefore, curled-leaf mutants are appealing genetic resources for breeding drought-tolerant cultivars [43]. Notably, the curling phenotype was present throughout the full growth period, irrespective of the moisture and temperature, unlike the recessive pepper *flc* mutant in which leaves are flat at night or under appropriate moisture and curled during the day [19]. This difference suggests that we have isolated novel alleles that affect leaf curling which is required further study to confirm.

The M_2_ population also included mutant lines with defects in inflorescence development or without reproductive growth, suggesting that the genes controlling flower induction might be mutated in these individuals. The *AGAMOUS* mutants are well characterized flower mutants in Arabidopsis [44], and we identified *agamous-like* mutants with inheritance stability. We selfed those M_2_ lines and observed a 3:7 segregation ratio of mutant to wild type in the M_3_ generation (data not shown). These mutant lines will be useful resources in flower biology and organ development-related research. We also identified several mutant lines that were male sterile. Male sterility is often induced by mutagenesis and is a desirable trait for breeding hybrids [45,46]. However, cytoplasmic male sterility is induced only rarely in crops such as sugar beet and pearl millet [47,48]. Test crosses and allelism assays will be needed to define the type of male sterility obtained here.

Fruit shape is critical for the market value of a horticultural commodity. Mutants with changes in fruit shape can lead to breeding profitable new cultivars according to market demand. We characterized several mutants with round, oval, cylindrical, and pyramid-shaped fruits. Fruit shape is challenging to measure, and it is a quantitative trait in nature. Additionally, the mechanisms controlling the fruit orientation, size, and shape remain elusive in pepper [4,22]. The mutants reported here will be useful for genetic studies aimed at uncovering the mechanisms that govern fruit shape-related traits. Fruit color is another essential trait for the value of commercial pepper cultivars. We identified a line with orange fruits that had stable inheritance, and a recent study employed this mutant to investigate fruit color variation in pepper [49]. Thus, the mutants identified here can serve as a primary material for identifying and characterizing the genes that regulate fruit color and morphology.

Many mutant studies in crops involve chemical mutagenesis-based TILLING experiments. Chemical mutagenesis, including EMS, generally produce single point mutations. Mutation densities among diploids have varied from 1 mutation per 150 kb to over 1 mutation per 1 million bp [50,51]. In our TILLING experiment, we analyzed the *eIF4E* gene with different primer sets. We discovered nine induced point mutations across the sets of chemically mutagenized populations. EMS converts GC to AT due to recurrent alkylation of guanine remnants [52]. In major plant model species, including Arabidopsis, wheat, maize, and pea, more than 99% of the mutations detected were GC:AT transitions [53]. We also detected point mutations that led to GC:AT base conversions here. Recently, TILLING methods are undergoing a series of adjustments to allow incorporation with high-throughput next-generation sequencing (NGS) technologies. Advanced NGS platforms like Illumina, SOL-iD, Ion Torrent, and PacBio and the steady reduction in sequencing costs has also contributed to the combination of NGS and TILLING methods. This approach can now be used efficiently in grains and horticultural crops [54,55,56]. Hence, the mutant population developed here can be analyzed by TILLING and sequencing, allowing the fast and robust detection of mutant alleles for use in pepper breeding in the near future.

## 5. Conclusions

Here, we employed EMS to generate genetic variability in a dwarf and compact pepper germplasm, Micro-Pep. We observed and characterized M_1_, M_2_, and M_3_ mutant phenotypes in plant growth and habit, leaf color and morphology, flower characteristics, and fruit color and morphology. Two of the phenotypes showed stable inheritance up to the M_3_ generation. With TILLING, we discovered nine putative point mutations in the *eIF4E* gene, four of which were confirmed by sequencing. Our Micro-Pep EMS mutant population showed great genetic diversity, making it a valuable resource for reverse genetic studies. These include TILLING approaches to determine the genetic factors underlying the phenotypes observed. In addition, availability of the pepper genome will facilitate map-based cloning of mutations of interest. Furthermore, newly developed strategies based on whole-genome sequencing can also be exploited. We have submitted our phenotypic data for the mutant population to an online browser (https://phenome-networks.com/) that is freely accessible to the scientific community. Finally, the mutants will be exchanged and shared among pepper breeders and researchers.

## Figures and Tables

**Table 1:** Primers used to amplify the *eIF4E* gene

Primer	Sequence (5ʹ->3ʹ)
Pvr1_Exon1_F	AAAGCACACAGCACCAACAA
Pvr1_Exon1_R	CAACCATAAATATACCCCGAGAA
Pvr1_Exon2-3_1F	GCGCTTTGAGTTATCGTACA
Pvr1_Exon2-3_1R	AGAGTGAGGCGAAGTTTGAG
Pvr1_Exon2-3_2F	GAGTTATCGTACAACTTGGACTGTG
Pvr1_Exon2-3_2R	GATATAACTGATGACTTCCCCTCTT
Pvr1_Exon4-5_F	GGTGATGTCGTTTTGTCTCA
Pvr1_Exon4-5_R	TCTGTGATTTGATGTGTGCTC

F, forward; R, reverse.

**Table 2:** Germination and survival percentage of the M_1_ generation

Treatments	Total No. of Seeds	Germinated Seeds	Seed Germination (%)	Surviving Seedlings	Surviving up to Seed Harvest
EMS	9500	6620	70%	4210	3996
Control	200	175	87%	165	157

**Table 3:** Growth pattern changes observed in the M_1_ generation

Categories	Number of Plants	Percentage (%)
Plant height (tall ^v^)	45	1.12%
Plant height (dwarf ^w^)	18	0.51%
Albino ^x^	23	0.61%
No flower ^y^	231	5.81%
Sterile ^z^	41	1.02%

^v^ >17 cm, ^w^ <10 cm, ^x^ Chlorotic leaves, ^y^ No inflorescence observed, ^z^ No functional or viable pollen were observed.

**Table 4:** Incidence of phenotypic variants observed among 13,000 M_2_ individuals.

Class	Sub-Class	Description	No. of Mutant Lines	Rate (%)	Segregation Mutant: Wild Type *
Plant growth	Tall	>18 cm	37	9.9	3:7
	Dwarf	<10 cm	7	1.9	3:7
	Retarded growth	No visible stem	15	4.0	3:7
	Abnormal branching	Spreading multi-whorled canopy	48	12.9	3:7
Leaf	Color	Mottled pale-green	18	4.8	2:8
		Light-green	6	1.6	3:7
		Mottled-yellow	7	1.9	2:8
		Dark-green	13	3.5	3:7
		Pale-green	29	7.8	3:7
		Silver-green	3	0.8	1:9
	Morphology	Leathery texture	8	2.2	2:8
		Undulation	5	1.3	2:8
		Upward curling	7	1.1	3:7
		Downward curling	4	1.9	3:7
		Bushy	3	0.8	2:8
		Obovate shape	2	0.5	1:9
		Long petiole	22	5.9	3:7
		Fused	1	0.3	2:8
		Prolific	11	3.0	2:8
		Large size	19	5.1	3:7
		Scabrous	12	3.2	1:9
		Narrow	9	2.4	1:9
Flower	Inflorescence	Flowerless	27	7.3	4:6
		Twin flower	3	0.8	1:9
		Sterile	21	5.6	2:8
	Organ color	Pink anther	1	0.3	1:9
	Morphology	Filamentous stamen	1	0.3	3:7
		AGAMOUS type	4	1.1	3:7
		Fasciculation type	1	0.3	2:8
		Swelled ovary with short stamen	3	0.8	2:8
		Shell type	1	0.3	1:9
Fruits	Color	Orange mature color	7	1.9	2:8
		Two different mature colors (red and yellow)	1	0.3	2:8
	Morphology	Round shape	5	1.3	3:7
		Cylindrical shape	2	0.5	1:9
		Oval shape	3	0.8	2:8
		Multiple fruit on a single calyx	1	0.3	1:9
		Pyramid shape	4	1.1	2:8
		Two shapes on one plant	1	0.3	2:8
Total			372	100	

* The number of individuals observed with the wild-type or mutant phenotype in the M_2_ mutant lines.

**Table 5:** Summary of EMS-induced pepper mutant screening for the *eIF4E* gene by TILLING

Mutant	Position of Mutation Detection	Mutant Population	Mutation Confirmed by Sanger Sequencing	Mutation Type
173	Pvr1_1Exon1	Micro-Pep	No variation	-
P1B8	Pvr1_1Exon1	Yuwol-cho	546th bp	Intron mutation
P4A7	Pvr1_1Exon1	Yuwol-cho	628th bp	Intron mutation
P4B4	Pvr1_1Exon1	Yuwol-cho	504th bp	Intron mutation
P4B11	Pvr1_1Exon1	Yuwol-cho	No variation	-
P1C3	Pvr1_2-3Exon2-3	Yuwol-cho	No variation	-
P2E3	Pvr1_2-3Exon2-3	Yuwol-cho	No variation	-
P6F6	Pvr1_2-3Exon2-3	Micro-Pep	No variation	-
P3C10	Pvr1_4-5Exon4-5	Yuwol-cho	5261st bp	Intron mutation

## References

[1] Bosland P.W., Votava E.J., Votava E.M. (2012). Peppers: Vegetable and Spice Capsicums.

[2] Siddique M.I., Lee H.Y., Ro N.Y., Han K., Venkatesh J., Solomon A.M., Patil A.S., Changkwian A., Kwon J.K., Kang B.C. (2019). Identifying candidate genes for *Phytophthora capsici* resistance in pepper (*Capsicum annuum*) via genotyping-by-sequencing-based QTL mapping and genome-wide association study. Sci. Rep..

[3] Jeong H.J., Kwon J.K., Pandeya D., Hwang J., Hoang N.H., Bae J.H., Kang B.C. (2012). A survey of natural and ethyl methane sulfonate-induced variations of *eIF4E* using high-resolution melting analysis in *Capsicum*. Mol. Breed..

[4] Jo Y.D., Kim S.H., Hwang J.E., Kim Y.S., Kang H.S., Kim S.W., Kwon S.J., Ryu J., Kim J.B., Kang S.Y. (2016). Construction of mutation populations by gamma-ray and carbon beam irradiation in chili pepper (*Capsicum annuum* L.). Hortic. Environ. Biotechnol..

[5] Lee H.Y., Ro N.Y., Jeong H.J., Kwon J.K., Jo J., Ha Y., Jung A., Han J.W., Venkatesh J., Kang B.C. (2016). Genetic diversity and population structure analysis to construct a core collection from a large Capsicum germplasm. BMC Genet..

[6] Solomon A.M., Han K., Lee J.H., Lee H.Y., Jang S., Kang B.C. (2019). Genetic diversity and population structure of Ethiopian *Capsicum* germplasms. PLoS ONE.

[7] Hwang D., Jeong H.J., Kwon J.K., Kim H., Kang S.Y., Kang B.C. (2014). Phenotypic variants among ethyl methanesulfonate M 2 mutant lines in *Capsicum annuum*. Plant Genet. Resour..

[8] Espina M.J., Ahmed C.M., Bernardini A., Adeleke E., Yadegari Z., Arelli P., Pantalone V., Taheri A. (2018). Development and phenotypic screening of an ethyl methane sulfonate mutant population in soybean. Front. Plant Sci..

[9] Voytas D.F., Gao C. (2014). Precision genome engineering and agriculture: Opportunities and regulatory challenges. PLoS Biol..

[10] Lu S., Yin X., Spollen W., Zhang N., Xu D., Schoelz J., Bilyeu K., Zhang Z.J. (2015). Analysis of the siRNA-mediated gene silencing process targeting three homologous genes controlling soybean seed oil quality. PLoS ONE.

[11] Daskalov S. (1986). Mutation breeding in pepper. IAEA.

[12] Jo Y.D., Kim J.B. (2019). Frequency and spectrum of radiation-induced mutations revealed by whole-genome sequencing analyses of plants. Quantum Beam Sci..

[13] Talebi A.B., Talebi A.B., Shahrokhifar B. (2012). Ethyl methane sulphonate (EMS) induced mutagenesis in Malaysian rice (cv. MR219) for lethal dose determination. Am. J. Plant Sci.

[14] Arisha M.H., Liang B.K., Shah S.N.M., Gong Z.H., Li D.W. (2014). Kill curve analysis and response of first generation *Capsicum annuum* L. B12 cultivar to ethyl methane sulfonate. Genet. Mol. Res..

[15] Piron F., Nicolai M., Minoia S., Piednoir E., Moretti A., Salgues A., Zamir D., Caranta C., Bendahmane A. (2010). An induced mutation in tomato eIF4E leads to immunity to two potyviruses. PLoS ONE.

[16] Gauffier C., Lebaron C., Moretti A., Constant C., Moquet F., Bonnet G., Caranta C., Gallois J.L. (2016). A TILLING approach to generate broad-spectrum resistance to potyviruses in tomato is hampered by eIF4E gene redundancy. Plant J..

[17] Xi-ou X., Wenqiu L., Wei L., Xiaoming G., Lingling L., Feiyue M., Yuge L. (2017). The analysis of physiological variations in M2 generation of *Solanum melongena* L. Mutagenized by ethyl methane sulfonate. Front. Plant Sci..

[18] Jabeen N., Mirza B. (2002). Ethyl methane sulfonate enhances genetic variability in *Capsicum annuum*. Asian J. Plant Sci..

[19] Bosland P.W. (2002). Inheritance of a novel flaccid mutant in *Capsicum annuum*. J. Hered..

[20] Paran I., Borovsky Y., Nahon S., Cohen O. (2007). The use of induced mutations to study shoot architecture in Capsicum. Isr. J. Plant Sci..

[21] Arisha M.H., Shah S.N.M., Gong Z.H., Jing H., Li C., Zhang H.X. (2015). Ethyl methane sulfonate induced mutations in M2 generation and physiological variations in M1 generation of peppers (*Capsicum annuum* L.). Front. Plant Sci..

[22] Chaim A.B., Borovsky Y., Rao G.U., Gur A., Zamir D., Paran I. (2006). Comparative QTL mapping of fruit size and shape in tomato and pepper. Isr. J. Plant Sci..

[23] Oleykowski C.A., Bronson Mullins C.R., Godwin A.K., Yeung A.T. (1998). Mutation detection using a novel plant endonuclease. Nucleic Acids Res..

[24] Slade A.J., Fuerstenberg S.I., Loeffler D., Steine M.N., Facciotti D. (2005). A reverse genetic, nontransgenic approach to wheat crop improvement by TILLING. Nat. Biotechnol..

[25] Till B.J., Reynolds S.H., Greene E.A., Codomo C.A., Enns L.C., Johnson J.E., Burtner C., Odden A.R., Young K., Taylor N.E. (2003). Large-scale discovery of induced point mutations with high-throughput TILLING. Genome Res..

[26] Henikoff S., Till B.J., Comai L. (2004). TILLING. Traditional mutagenesis meets functional genomics. Plant Physiol..

[27] Page D.R., Grossniklaus U. (2002). The art and design of genetic screens: Arabidopsis thaliana. Nat. Rev. Genet..

[28] Østergaard L., Yanofsky M.F. (2004). Establishing gene function by mutagenesis in Arabidopsis thaliana. Plant J..

[29] Menda N., Semel Y., Peled D., Eshed Y., Zamir D. (2004). In silico screening of a saturated mutation library of tomato. Plant J..

[30] Martí E., Gisbert C., Bishop G.J., Dixon M.S., García-Martínez J.L. (2006). Genetic and physiological characterization of tomato cv. Micro-Tom. J. Exp. Bot..

[31] Gady A.L.F., Hermans F.W.K., Van de Wal M.H.B.J., van Loo E.N., Visser R.G.F., Bachem C.W.B. (2009). Implementation of two high through-put techniques in a novel application: Detecting point mutations in large EMS mutated plant populations. Plant Methods.

[32] Minoia S., Petrozza A., D’Onofrio O., Piron F., Mosca G., Sozio G., Cellini F., Bendahmane A., Carriero F. (2010). A new mutant genetic resource for tomato crop improvement by TILLING technology. BMC Res. Notes.

[33] Shah S.N., Gong Z.H., Arisha M.H., Khan A., Tian S.L. (2015). Effect of ethyl methyl sulfonate concentration and different treatment conditions on germination and seedling growth of the cucumber cultivar Chinese long (9930). Genet. Mol. Res..

[34] Sikder S., Biswas P., Hazra P., Akhtar S., Chattopadhyay A., Badigannavar A.M., D’Souza S.F. (2013). Induction of mutation in tomato (*Solanum lycopersicum* L.) by gamma irradiation and EMS. Indian J. Genet. Plant Breed..

[35] Alcantara T.P., Bosland P.W., Smith D.W. (1996). Ethyl methanesulfonate-induced seed mutagenesis of *Capsicum annuum*. J. Hered..

[36] Roychowdhury R., Tah J. (2013). Mutagenesis—A potential approach for crop improvement. Crop Improvement.

[37] Saito T., Ariizumi T., Okabe Y., Asamizu E., Hiwasa-Tanase K., Fukuda N., Mizoguchi T., Yamazaki Y., Aoki K., Ezura H. (2011). TOMATOMA: A novel tomato mutant database distributing Micro-Tom mutant collections. Plant Cell Physiol..

[38] Lippert L.F., Bergh B.O., Cook A.A. (1964). Three variegated seedling mutants in the pepper: Multiple allelism indicated in crossing studies. J. Hered..

[39] Fridborg I., Kuusk S., Moritz T., Sundberg E. (1999). The Arabidopsis dwarf mutant shi exhibits reduced gibberellin responses conferred by overexpression of a new putative zinc finger protein. Plant Cell.

[40] Wang D., Bosland P.W. (2006). The genes of Capsicum. HortScience.

[41] Wu Z., Zhang X., He B., Diao L., Sheng S., Wang J., Guo X., Su N., Wang L., Jiang L. (2007). A chlorophyll-deficient rice mutant with impaired chlorophyllide esterification in chlorophyll biosynthesis. Plant Physiol..

[42] Coschigano K.T., Melo-Oliveira R., Lim J., Coruzzi G.M. (1998). Arabidopsis gls mutants and distinct Fd-GOGAT genes: Implications for photorespiration and primary nitrogen assimilation. Plant Cell.

[43] Murchie E.H., Chen Y.Z., Hubbart S., Peng S., Horton P. (1999). Interactions between senescence and leaf orientation determine in situ patterns of photosynthesis and photoinhibition in field-grown rice. Plant Physiol..

[44] Yanofsky M.F., Ma H., Bowman J.L., Drews G.N., Feldmann K.A., Meyerowitz E.M. (1990). The protein encoded by the Arabidopsis homeotic gene agamous resembles transcription factors. Nature.

[45] Shifriss C. (1997). Male sterility in pepper (*Capsicum annuum* L.). Euphytica.

[46] Siddique M.I., Wai K.P.P., Mo H.S., Yoo H.J., Jang K.S., Jeon S.G., Hwang J.E., Kim B.S. (2017). Resistance to *Phytophthora capsici*, restorer-of-fertility genotype for cytoplasmic male sterility and chemical quality components of breeding lines developed for improvement of subicho, a land race of pepper in Yeongyang. Hort. Sci. Technol..

[47] Burton G.W., Hanna W.W. (1982). Stable cytoplasmic male-sterile mutants induced in Tift 23DB1 pearl millet with mitomycin and streptomycin 1. Crop Sci..

[48] Kinoshita T., Takahashi M.E., Mikami T. (1982). Cytoplasmic mutation of male sterility induced by chemical mutagens in sugar beets. Proc. Jpn. Acad. Ser. B.

[49] Jeong H.B., Kang M.Y., Jung A., Han K., Lee J.H., Jo J., Lee H.Y., An J.W., Kim S., Kang B.C. (2019). Single-molecule real-time sequencing reveals diverse allelic variations in carotenoid biosynthetic genes in pepper (*Capsicum spp*.). Plant Biotechnol. J..

[50] Till B.J., Zerr T., Comai L., Henikoff S. (2006). A protocol for TILLING and Ecotilling in plants and animals. Nat. Protoc..

[51] Amri-Tiliouine W., Laouar M., Abdelguerfi A., Jankowicz-Cieslak J., Jankuloski L., Till B.J. (2018). Genetic variability induced by gamma rays and preliminary results of low-cost TILLING on M_2_ generation of Chickpea (*Cicer arietinum* L.). Front. Plant Sci..

[52] Sega G.A. (1984). A review of the genetic effects of ethyl methanesulfonate. Mutat. Res. Rev. Genet. Toxicol..

[53] Kurowska M., Daszkowska-Golec A., Gruszka D., Marzec M., Szurman M., Szarejko I., Maluszynski M. (2011). TILLING-a shortcut in functional genomics. J. Appl. Genet..

[54] Tsai H., Howell T., Nitcher R., Missirian V., Watson B., Ngo K.J., Lieberman M., Fass J., Uauy C., Tran R.K. (2011). Discovery of rare mutations in populations: TILLING by sequencing. Plant Physiol..

[55] Guo Y., Abernathy B., Zeng Y., Ozias-Akins P. (2015). TILLING by sequencing to identify induced mutations in stress resistance genes of peanut (*Arachis hypogaea*). BMC Genom..

[56] Gupta P., Reddaiah B., Salava H., Upadhyaya P., Tyagi K., Sarma S., Datta S., Malhotra B., Thomas S., Sunkum A. (2017). Next-generation sequencing (NGS)-based identification of induced mutations in a doubly mutagenized tomato (*Solanum lycopersicum*) population. Plant J..

